# Serum Neurofilament Levels in Children With Febrile Seizures and in Controls

**DOI:** 10.3389/fnins.2020.579958

**Published:** 2020-09-29

**Authors:** Katrina S. Evers, Melanie Hügli, Sotirios Fouzas, Severin Kasser, Christian Pohl, Benjamin Stoecklin, Luca Bernasconi, Jens Kuhle, Sven Wellmann

**Affiliations:** ^1^Division of Neonatology and University of Basel Children’s Hospital (UKBB), Basel, Switzerland; ^2^Paediatric Respiratory Unit and Department of Neonatology, University Hospital of Patras, Patras, Greece; ^3^Neonatal Intensive Care Unit, Perth Children’s and King Edward Memorial Hospitals, Perth, WA, Australia; ^4^Institute of Laboratory Medicine, Kantonsspital Aarau, Aarau, Switzerland; ^5^Neurologic Clinic and Policlinic, Departments of Medicine, Biomedicine and Clinical Research, University Hospital Basel, University of Basel, Basel, Switzerland; ^6^Division of Neonatology, University Children’s Hospital Regensburg (KUNO), University of Regensburg, Regensburg, Germany

**Keywords:** neuronal biomarker, convulsion, epilepsy, neurofilament, paroxysmal

## Abstract

**Objective:**

Neuroaxonal damage is reflected by serum neurofilament light chain (sNfL) values in a variety of acute and degenerative diseases of the brain. The aim of this study was to investigate the impact of febrile and epileptic seizures on sNfL, serum copeptin, and prolactin levels in children compared with children with febrile infections without convulsions.

**Methods:**

A prospective cross-sectional study was performed in children aging 6 months to 5 years presenting with fever (controls, *n* = 61), febrile seizures (FS, *n* = 78), or epileptic seizures (ES, *n* = 16) at our emergency department. sNfL, copeptin, and prolactin were measured within a few hours after the event in addition to standard clinical, neurophysiological, and laboratory assessment. All children were followed up for at least 1 year after presentation concerning recurrent seizures.

**Results:**

Serum copeptin values were on average 4.1-fold higher in FS and 3.2-fold higher in ES compared with controls (both *p* < 0.01). Serum prolactin values were on average 1.3-fold higher in FS compared with controls ( *p* < 0.01) and without difference between ES and controls. There was no significant difference of mean sNfL values (95% CI) between all three groups, FS 21.7 pg/ml (19.6–23.9), ES 17.7 pg/ml (13.8–21.6), and controls 23.4 pg/ml (19.2–27.4). In multivariable analysis, age was the most important predictor of sNfL, followed by sex and C reactive protein. Neither the duration of seizures nor the time elapsed from seizure onset to blood sampling had an impact on sNfL. None of the three biomarkers were related to recurrent seizures.

**Significance:**

Serum neurofilament light is not elevated during short recovery time after FS when compared with children presenting febrile infections without seizures. We demonstrate an age-dependent decrease of sNfL from early childhood until school age. In contrast to sNfL levels, copeptin and prolactin serum levels are elevated after FS.

## Introduction

Febrile seizures (FS) are the most common convulsive events in children aged between 6 months and 5 years and arise in 2 to 5% of all children. FS are defined as seizures occurring during childhood associated with fever that is not caused by an infection of the central nervous system (Subcommittee on Febrile Seizures American Academy of Pediatrics, 2011). FS are classified as simple or complex seizures depending on age at onset, duration, short-term recurrence, and type of seizure (Livingston et al., 1979). In approximately one third of children with a first febrile seizure, a second episode, and in around 10%, three or more FS will occur (Berg et al., 1997). Especially prolonged FS may be associated with substantial long-term neurological morbidities such as temporal lobe epilepsy or mesial temporal sclerosis with possible subsequent intellectual disability (Pujar et al., 2018).

Prolactin is a polypeptide hormone secreted by the anterior pituitary gland but also in other tissues and organs such as adipose tissue, uterus, and immune cells. Apart from the production of milk, prolactin is also known to play a role in the regulation of the immune system, behavior, and metabolism. Initially, Trimble et al. debated that seizures could raise prolactin levels (Trimble, 1978). In the past decades, it has gained recognition in the support of the diagnosis of epileptic seizures in particular for the differentiation of generalized tonic–clonic or complex partial seizures from psychogenic non-epileptic seizures among adults and older children especially when the clinical setting does not provide video-EEG recording (Chen et al., 2005; Abubakr and Wambacq, 2016; Fisher, 2016).

Another hormone released by the pituitary gland is arginine vasopressin (AVP) which plays a major role not only in maintaining the fluid balance and vascular tonus but also in the regulation of the endocrine stress response. Copeptin derives from the same precursor molecule, is more stable, and is released into the periphery in the same ratio as AVP (Evers and Wellmann, 2016). Published data suggest that copeptin is involved in the thermoregulatory response to fever and convulsions and copeptin has lately been shown to have high diagnostic accuracy in FS (Kasting et al., 1980, 1981; Landgraf et al., 1990; Stocklin et al., 2015).

Neurofilaments (Nf) are highly specific major scaffolding proteins of neurons consisting of four subunits: the triplet of NfL (Nf light), Nf medium, and NfH (Nf heavy) chains and alpha-internexin in the CNS, or peripherin in the peripheral nervous system (Teunissen and Khalil, 2012). Disruption of the axonal cell membrane due to acute or chronic neuronal damage releases Nf into the interstitial fluid and eventually to the cerebrospinal fluid (CSF) and the blood compartment (Khalil et al., 2018).

Matsushige and colleagues recently determined serum pNF-H (phosphorylated form of neurofilament-heavy chain) levels in patients with prolonged and simple FS to evaluate neuronal damage and were able to show that serum pNF-H levels in children with prolonged FS were significantly higher than in children without FS (Matsushige et al., 2012). Shahim et al. (2013) demonstrated that CSF NfL levels in children were increased in status epilepticus compared with unspecified epilepsy and that NfL levels were significantly higher in lysosomal and mitochondrial disorders than in neurodegenerative disorders without known etiology. Higher NfL levels in children with suspected multiple sclerosis are predictive for clinically definite multiple sclerosis diagnosis (van der Vuurst de Vries et al., 2018; Wong et al., 2019). Furthermore, CSF NfL levels had the highest capability to distinguish opsoclonus–myoclonus syndrome from controls compared with other brain cell–specific biomarkers in a pediatric cohort (Pranzatelli et al., 2014).

The aims of this study were (1) to evaluate the short-term impact of convulsions on serum NfL (sNfL) levels in a cohort of children presenting with FS in comparison with children with febrile infections and epileptic seizures at an emergency department (ED); (2) to compare sNfL levels with other postictal serum biomarkers, namely, copeptin and prolactin; and (3) to characterize sNfL levels in the population of young children in general.

## Materials and Methods

The study was based on data and blood samples prospectively collected from a child cohort established at the University Children’s Hospital of Basel (UKBB), Switzerland, between May 2013 and November 2015. The Cantonal Ethics Committee of Basel approved the study protocol (EK352/12), and written informed consent was obtained from the parents. The study was registered in the clinical trial registry ClinicalTrials.gov (No. NCT01884766). Information concerning eligibility criteria and the inclusion procedure can be obtained elsewhere (Stocklin et al., 2015). Serum concentrations of NfL were determined with a Simoa assay, which was established using the NF-light assay ELISA kit from UmanDiagnostics (Umeå, Sweden), transferred onto the Simoa platform with a homebrew kit (Quanterix, Boston, MA, United States), and has been described in detail by our group previously (Disanto et al., 2017). Calibrators (neat) and serum samples (1:4 dilution) were measured in duplicates. Bovine lyophilized NfL was obtained from UmanDiagnostics. Calibrators ranged from 0 to 2000 pg/ml. Batch prepared calibrators were stored at −80°C. Intra- and interassay variabilities of the assay were <10%. Repeated measuring was performed for the few samples with intra-assay coefficients of variation >20%.

Measurement of copeptin levels was done in a batch analysis with a commercial sandwich immunofluorescence assay (B⋅R⋅A⋅H⋅M⋅S Copeptin proAVP; Thermo Fisher Scientific, Hennigsdorf/Berlin, Germany) as described in detail elsewhere (Morgenthaler et al., 2006). The lower detection limit of the copeptin assay was 0.69 pmol/L, and the functional assay sensitivity was <1 pmol/L.

Prolactin quantification was performed using the Roche Modular E 170 (Roche Diagnostics AG, Rotkreuz, Switzerland). The lower detection limit was 1 mU/L, and the inter-assay precision <3% coefficient of variance at 102, 450, and 816 mU/L, respectively.

### Statistics

Statistical analyses were performed using SPSS for Windows version 24 (IBM, United States) and included descriptive statistics, Spearman’s rank-order correlation analyses, and multiple linear regressions (MLR) using sNfL as dependent variable. sNfL variables were log10 transformed for the correlations and MLR. The independent variables included for MLR were based on significant correlations and significant non-parametric univariate analyses such as the one-way ANOVA test (with Bonferroni correction for multiple comparisons), Mann–Whitney *U*–test (2 levels), Kruskal–Wallis test (>2 levels), χ^2^ test, or Fisher’s exact test. The discriminatory ability of both copeptin and prolactin was assessed by receiver operating characteristic (ROC) curve analysis and was compared by means of the area under the curve (AUC). A *p*-value of <0.05 was considered statistically significant.

## Results

**FIGURE 1:**
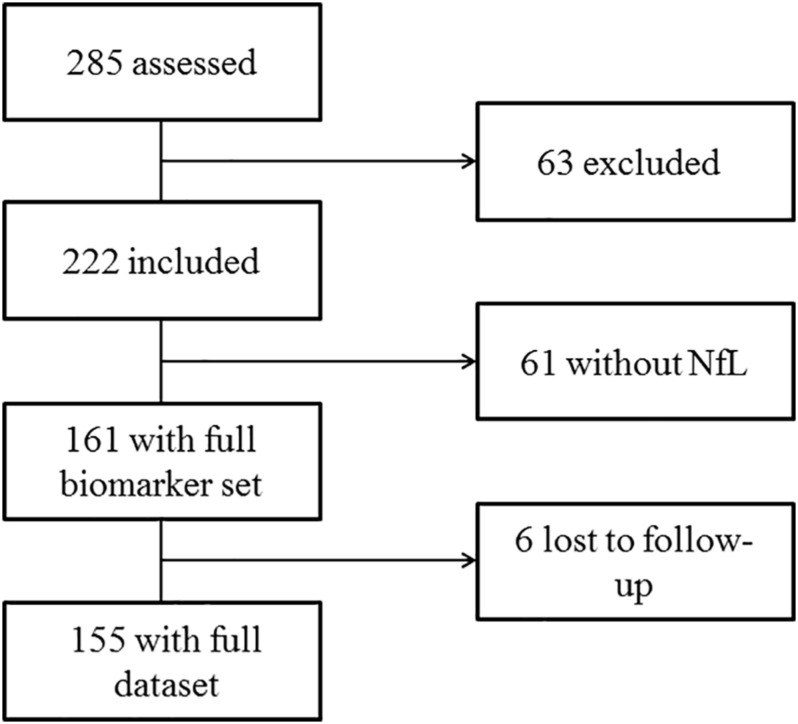
Consort flow diagram.

We recruited a total of 285 children from May 2013 until November 2015. After exclusion of 63 infants, a total of 222 children were included in the final analysis. Of these, 61 did not have enough material for the analysis of sNfL, resulting in complete biomarker sets of 161 children. Six children were lost to follow-up (Figure 1). The children’s age varied between 6 and 163 months; 44% were female. We allocated 78 children to the FS group, 16 to the ES group, and 61 febrile children without seizures were defined as controls. The characteristics of all groups are presented in Table 1.

**TABLE 1:** Characteristics of the study groups.

	Controls (*n* = 61)	Febrile seizures (*n* = 78)	Epileptic seizures (*n* = 16)
Males/females	33/28	43/35	10/6
Age, months	29.4 ± 17.8 (6–72)	24.8 ± 14.5 (6–63)	53.9 ± 45.8 (9–163)*
Body weight, kg	12.9 ± 4.2 (6.8–27)	12.1 ± 3.5 (6.0–23.0)	18.4 ± 13.0 (4.4–56)^†^
History of seizures	NA	16 (20.5)	9 (56.3)^‡^
Temperature at home, °C	39.6 ± 0.7 (37.7–41.3)	39.3 ± 0.6 (38.0–41.0)	NA
Temperature at ED, °C	38.3 ± 1.0 (36.0–40.5)	38.6 ± 0.8 (36.5–40.1)	NA
Duration of event, min	NA	6.5 ± 8.1 (1–40)	5.1 ± 5.2 (1–20)
Time to presentation, min	NA	107 ± 70.7 (1–330)	96.2 ± 60.9 (7–240)§
Laboratory data at ED			
Hct, %	35.6 ± 3.7 (27.3–43.3)	37.2 ± 4.2 (28.9–56.0)	38.2 ± 3.3§§(31.8–42.3)§
WBC × 1000/mm^3^	12.4 ± 7.2 (1.9–40.8)	12.9 ± 7.1 (3.4–34.2)	8.6 ± 2.7 (5.2–14.7)
Na, mmol/L	136.1 ± 3.2 (129–142)	135 ± 2.9 (118–141)	138 ± 2.1 (135–143)
Cl, mmol/L	105 ± 3.2 (98–112)	105 ± 2.6 (98–112)	106 ± 2.2 (101–110)
pH	7.37 ± 0.05 (7.20–7.40)	7.36 ± 0.06 (7.20–7.50)	7.27 ± 0.07** (7.10–7.30)**
CO_2_, mmHg	31.6 ± 4.4^†^^†^ (21–41)^†^^†^	33 ± 5.1 (24–54)	43.2 ± 10.7 (34–70)
Bicarbonate, mmol/L	21.7 ± 2.6 (13.9–26.1)	21.6 ± 1.6 (17.5–25.3)	21.5 ± 2.6 (14.9–25.2)
Lactate, mmol/L	1.5 ± 0.8 (0.9–4.5)	1.5 ± 0.7 (0.7–4.5)	1.2 ± 0.5 (0.6–2.2)
CRP, mg/dl	50.5 ± 50.2 (0.3–220)^†^^†^	12.6 ± 18.6 (0.3–91)	1.3 ± 2.4 (0.3–8.0)

*Data are presented as mean ± SD (range) unless stated otherwise. ^∗^*p* = 0.042 vs controls and *p* = 0.002 vs febrile seizures. ^†^*p* = 0.043 vs. febrile seizures. ^‡^*p* = 0.010 vs febrile seizures. ^§^*p* = 0.755 vs febrile seizures. ^§§^*p* = 0.039 vs controls. ^¶^*p* = 0.002 vs febrile seizures. ^∗∗^*p* < 0.001 vs febrile seizures and controls. ^†⁣†^*p* < 0.001 vs febrile and epileptic seizures. Between-group comparisons were performed with Mann–Whitney *U*-test, Kruskal–Wallis one-way ANOVA test (with Bonferroni correction for multiple comparisons), χ^2^ test, or Fisher’s exact test, as appropriate. ED, emergency department; NA, not available; WBC, white blood cell.*

There was no significant difference in age, body weight, and temperature at home or at ED when comparing the controls with FS group; the ES group had overall slightly but not significantly higher values in age and body weight than the other groups (Table 1). Regarding the laboratory data, pH in the ES group was significantly lower compared with the FS and control groups, whereas the controls exhibited significantly higher C reactive protein (CRP) levels than the FS and ES groups (Table 1). In total, 16 children (20.5%) in the FS and nine children (56.3%) in the ES group had a history of previous convulsive events. Serum values of NfL, copeptin, and prolactin in the different study groups are summarized in Table 2. When comparing the biomarkers in accordance to presence of fever, mean sNfL levels (95% CI) were only slightly higher in children with fever than in children without fever [fever: 22.1 pg/ml (20.1–24.1), no fever: 21.6 pg/ml (17.8–25.4), *p* = 0.017]. The evaluation of impact of seizures on biomarker levels revealed that seizures did not affect the levels of sNfL [20.8 pg/ml (18.9–22.7) vs 23.6 pg/ml (19.5–27.7)], whereas prolactin was slightly elevated in children presenting with convulsions compared with children without seizures [415 mU/L (366–464) vs 320 mU/L (277–362)] and copeptin was significantly higher in the group with seizures compared with no seizures [37.0 pmol/L (26.0–48.0) vs 9.6 pmol/L (6.4–12.8), *p* < 0.001]. Of note, no differences were found between time to presentation, which is the time elapsed from event onset to presentation at the emergency department (Table 1). Because blood sampling was done in all patients with FS or ES upon presentation, there was also no difference in the time to sampling.

**TABLE 2:** Differences in biomarkers among study groups.

	Controls (*n* = 61)	Febrile seizures (*n* = 78)	Epileptic seizures (*n* = 16)
sNfL, pg/ml	23.4 (19.2–27.4)	21.7 (19.6–23.9)	17.7 (13.8–21.6)
Prolactin, mU/L	320 (277–362)^∗^	411 (365–458)^∗^	429 (266–592)
Copeptin, pmol/L	9.7 (6.4–12.9)^†,‡^	39.9 (26.1–53.8)^†^	30 (13.7–46.2)^‡^

*Data are presented as mean (CI). ^∗^*p* = 0.012 for febrile seizures vs controls. ^†^*p* < 0.001 for febrile seizures vs controls. ^‡^*p* = 0.002 for epileptic seizures vs controls. Between-group comparisons were performed with Kruskal–Wallis one-way ANOVA test (with Bonferroni correction for multiple comparisons).*

**FIGURE 2:**
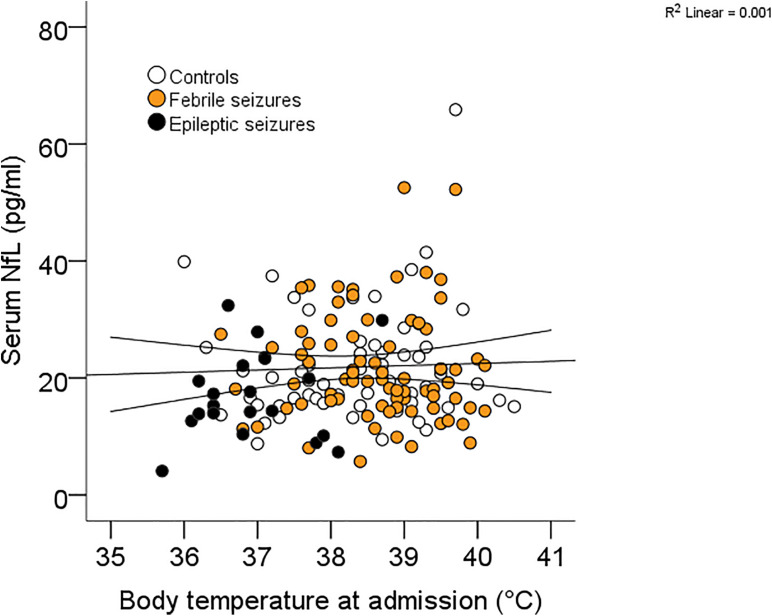
Correlation between sNfL and body temperature.

**FIGURE 3:**
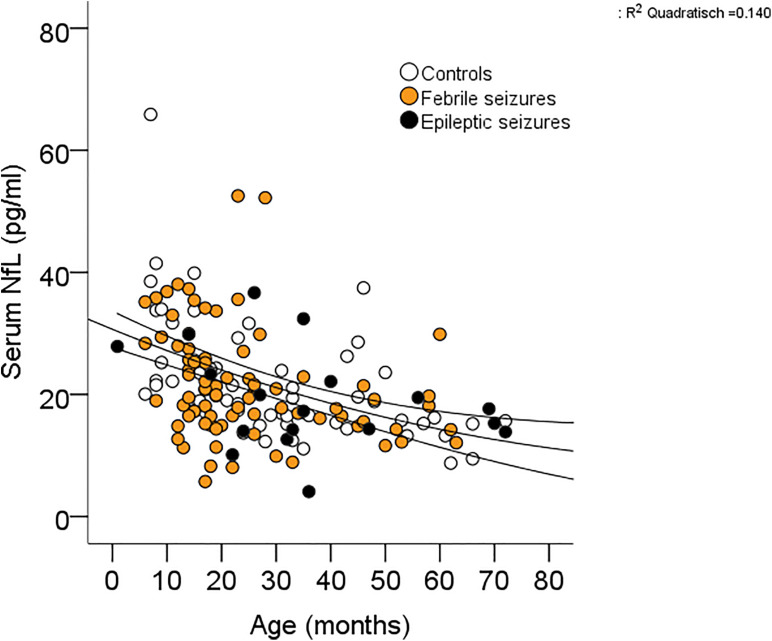
Correlation between sNfL and age.

Receiver operating characteristic curve analysis revealed that the ability to diagnose seizures differed clearly between the individual biomarkers (Table 3) with copeptin demonstrating the highest AUC levels compared with prolactin and sNfL [FS + ES vs controls: copeptin 0.804 (0.733–0.875) pmol/L; prolactin 0.620 (0.529–0.710) mU/L; sNfL 0.462 (0.370–0.555) pg/ml]. In consideration of the finding that sNfL levels were higher in the presence of fever, we had a closer look at the relationship between sNfL and fever and were not able to detect a correlation (Figure 2). With respect to the type of FS, we could not find any differences between simple and complex FS in biomarker levels [simple FS: sNfL: 20.9 (19.0–22.8) pg/ml; prolactin: 415 (366–464) mU/L, copeptin: 37.8 (26.5–49.1) pmol/L; complex FS: sNfL: 23.6 (18.9–28.4) pg/ml, prolactin: 425 (354–496) mU/L, copeptin: 38.6 (22.9–54.2) pmol/L]. When appointing sNfL as a dependent variable in univariate models, sNfL had a significant inverse relationship with age and body weight (Table 4), indicating an age-dependent decrease of sNfL from early childhood until school age (Figure 3). MLR revealed age as the most important predictor of sNfL, followed by male sex and CRP. After including the two other biomarkers copeptin and prolactin into a model, also copeptin turned out to be a strong predictor for sNfL.

**TABLE 3:** Ability of biomarkers to diagnose seizures.

	All seizures (FS + ES vs controls)	Febrile seizures (FS vs controls)
sNfL	0.462 (0.370–0.555)	0.494 (0.396–0.592)
Prolactin	0.620 (0.529–0.710)	0.648 (0.554–0.741)
Copeptin	0.804 (0.733–0.875)	0.807 (0.733–0.882)

*Data are AUC (95% CI). ES, epileptic seizures; FS, febrile seizures; sNfL, serum neurofilament light chain.*

**TABLE 4:** sNfL dependencies.

	Unadjusted effect			Adjusted effect			
	*R*^2^	Beta	*p*-value	Model 1 (*R*^2^ 0.201)		Model 2 (*R*^2^ 0.301)	
				Beta	*p*-value	Beta	*p*-value
Seizures	0.013	−0.114	0.159				
Male gender	0.001	−0.027	0.736	0.232	0.035	0.300	0.005
Age	**0.165**	−**0.406**	**<0.001**	−0.337	0.002	−0.375	0.001
Body weight	**0.139**	−**0.373**	**<0.001**				
Temperature at home	0.012	0.110	0.235				
Temperature at ED	0.007	0.086	0.288				
Hct	0.011	−0.107	0.195				
WBC	0.002	−0.050	0.566				
Na	0.005	−0.069	0.437				
Cl	0.001	0.002	0.979				
pH	**0.051**	**0.227**	**0.010**				
CO_2_	0.019	−0.138	0.121				
Bicarbonate	0.013	0.115	0.199				
Lactate	0.008	−0.088	0.327				
CRP	0.008	0.089	0.310	0.278	0.012	0.241	0.023
Prolactin	0.001	0.016	0.843				
Copeptin	**0.056**	−**0.237**	**0.003**			−0.318	0.003

*The unadjusted effect of each parameter was calculated by simple linear regression analysis using sNfL values (after logarithmic transformation) as the dependent variable. Significant (*p*-value < 0.05) parameters of the unadjusted effect are displayed in bold. The adjusted effect was calculated by stepwise linear regression analysis. CRP, C reactive protein; ED, emergency department; sNfL, serum neurofilament light chain; WBC, white blood cell.*

## Discussion

We prospectively investigated serum levels of NfL, copeptin, and prolactin in children presenting at an emergency department with FS, ES, or febrile infections without convulsions (controls). Our results provide evidence (1) that sNfL levels are not increased when measured within a few hours after convulsions in contrast to copeptin and prolactin levels; (2) that sNfL levels are higher in younger children, boys, and children with elevated CRP and elevated copeptin levels; and (3) that none of the three serum biomarkers are predictive for the recurrence of seizures.

The absent impact of convulsions on sNfL levels when measured a few hours after the events underlines the current state of evidence that simple FS are benign and do not increase the risk for the development of neurologic deficits (Steering Committee on Quality Improvement and Management, Subcommittee on Febrile Seizures American Academy of Pediatrics, 2008; Leaffer et al., 2013). Visser et al. (2012) also state that FS are not associated with problems in behavior or executive functioning in preschool children but did note an association of recurrent FS with an increased risk of expressive language delay at the age of 2.5 years which supports earlier findings about poorer language skills in school-aged children with a history of FS (Wallace, 1984). Matsushige et al. (2012) investigated the heavy chain of neurofilament (NfH) in serum of children suffering from febrile or epileptic seizures. The authors found a significant correlation between seizure duration and serum NfH levels during the first week in children with FS (Matsushige et al., 2012). Thus, whether sNfL levels may rise during recovery after febrile and epileptic convulsions warrants future studies.

Univariate analyses revealed a strong inverse relationship between sNfL and age and weight (Table 4 and Figure 3). In multivariate analysis, for which weight was removed due to collinearity with age, age had the greatest impact on sNfL followed by male sex and CRP levels independently of seizures and fever. A very similar age dependency was described recently in a cohort of neurologically healthy children with decreasing sNfL in older children (Khalil et al., 2020; Reinert et al., 2020). In addition, between the age of 10 and 15 years, sNfL levels appear to mark a nadir, and beyond youth, sNfL levels increase in a linear fashion until the age of about 60 years. Afterward, sNfL levels were reported to rise much steeper (Khalil et al., 2020; Reinert et al., 2020). Thus, considering sNfL level during the whole life cycle from high levels in newborn infants (Depoorter et al., 2018), decreasing until late childhood and then steadily increasing, sNfL levels represent a u-shaped curve. A possible explanation for high level in newborns is the developing brain with a high neuron turnover and a specialized system of tubulo-endoplasmic reticulum for protein transport. By the appearance of cerebral vessels being more fragile in infants than in adults, this might have the effect that the developing brain is more vulnerable (Saunders et al., 2012). In general, sNfL seems to reflect the substantial brain growth until adolescence followed by neuronal loss, which is associated with normal aging. Sexual disparity of biomarkers was described previously for copeptin in infants with higher levels in males. However, data on gender differences in sNfL are lacking (Burckhardt et al., 2014).

We observed that prolactin was elevated in the FS group when compared with the control group. The routine use of prolactin is not recommended due to limited accuracy. Moreover, copeptin levels were significantly higher in the FS group than in the control group and may be more useful for distinction of the underlying cause of the convulsive event (Stocklin et al., 2015; Pechmann et al., 2019). In contrast to these findings, our results could not provide additional support that copeptin and prolactin have the potential to predict upcoming convulsive events because none of the two biomarkers were related to recurrent seizures.

A few limitations need to be considered: the control group consisted of children presenting with febrile infections and our study revealed that sNfL levels are elevated in presence of fever alone and also correlate with CRP levels. This may lead to the suggestion that sNfL levels might be increased when compared with levels of healthy children without fever or contrariwise might only be elevated due to the rise in body temperature. We therefore propose to compare with a healthy afebrile cohort for verification of our hypothesis in potential upcoming studies. Furthermore, we must bear in mind that the diagnosis of a febrile seizure is solely based on the medical history and description of the caregivers; estimation by qualified personnel is therefore dependent on the statement of the accompanying parents. An overlap with simple shivering due to rise of temperature can therefore not be excluded. Besides, we merely analyzed blood samples at one timepoint; results of a further timepoint would give valuable information on the trend of sNfL levels and additionally might aid to assess the severity of suggested neuronal loss.

In conclusion, sNfL levels are not associated with febrile or epileptic seizures a few hours after the event, but significantly correlate with age, gender, and CRP. These findings are reassuring and indicate the benign nature of FS.

## Data Availability Statement

The raw data supporting the conclusions of this article will be made available by the authors, without undue reservation.

## Ethics Statement

The studies involving human participants were reviewed and approved by the Cantonal Ethics Committee of Basel. Written informed consent to participate in this study was provided by the participants’ legal guardian/next of kin.

## Author Contributions

SW, BS, KE, and JK conceived and designed the study. BS, SK, and CP were responsible for patient recruitment. JK and LB performed the biomarker measurements. SF performed the statistical analysis and prepared the tables and figures. SW, KE, MH, and JK interpreted the data. KE and SW drafted the initial manuscript. All authors critically revised the manuscript for important intellectual content, agreed on the final manuscript, and approved its submission for publication.

## Conflict of Interest

The authors declare that the research was conducted in the absence of any commercial or financial relationships that could be construed as a potential conflict of interest.
